# UV-grafted polyitaconic acid functionalization of PEEK promotes osteoimmunomodulation and osseointegration with PI3K-AKT-associated autophagy regulation

**DOI:** 10.1093/rb/rbag169

**Published:** 2026-08-05

**Authors:** Liping Zhou, Yang Zhou, Changyue Liu, Xiangyu Li, Chen Wu, Hanling Deng, Wenjiao Zeng, Yongheng Zhang, Ying Liu, Lvhua Liu, Ang Gao, Huaiyu Wang, Yanyan Zheng

**Affiliations:** School of Pharmacy and Institute of Pharmacy, North Sichuan Medical College, Nanchong 637000, China; Department of Stomatology, Affiliated Hospital of North Sichuan Medical College, Nanchong 637000, China; Department of Stomatology, North Sichuan Medical College, Nanchong 637000, China; Department of Stomatology, Affiliated Hospital of North Sichuan Medical College, Nanchong 637000, China; Department of Stomatology, North Sichuan Medical College, Nanchong 637000, China; Department of Stomatology, Affiliated Hospital of North Sichuan Medical College, Nanchong 637000, China; Department of Stomatology, North Sichuan Medical College, Nanchong 637000, China; Department of Stomatology, Affiliated Hospital of North Sichuan Medical College, Nanchong 637000, China; Department of Stomatology, North Sichuan Medical College, Nanchong 637000, China; School of Pharmacy and Institute of Pharmacy, North Sichuan Medical College, Nanchong 637000, China; School of Pharmacy and Institute of Pharmacy, North Sichuan Medical College, Nanchong 637000, China; Department of Stomatology, Affiliated Hospital of North Sichuan Medical College, Nanchong 637000, China; Department of Stomatology, North Sichuan Medical College, Nanchong 637000, China; Department of Stomatology, Affiliated Hospital of North Sichuan Medical College, Nanchong 637000, China; Department of Stomatology, North Sichuan Medical College, Nanchong 637000, China; School of Pharmacy and Institute of Pharmacy, North Sichuan Medical College, Nanchong 637000, China; Center for AI-Driven Medical Research, Shenzhen Institutes of Advanced Technology, Chinese Academy of Sciences, Shenzhen 518055, China; Center for AI-Driven Medical Research, Shenzhen Institutes of Advanced Technology, Chinese Academy of Sciences, Shenzhen 518055, China; School of Pharmacy and Institute of Pharmacy, North Sichuan Medical College, Nanchong 637000, China

**Keywords:** polyetheretherketone, surface modification, osseointegration, osteoimmunomodulation, itaconic acid

## Abstract

Polyetheretherketone (PEEK) is a promising orthopedic implant material because of its favorable mechanical properties and biocompatibility, yet its bioinert surface limits osseointegration. Here, we develop a polyitaconic acid-functionalized PEEK surface (PEEK-PITAc) through a one-step ultraviolet-induced graft polymerization strategy to improve its osteoimmunomodulatory performance. The PITAc-grafted PEEK surface exhibits enhanced hydrophilicity and bioactivity while maintaining its original morphology. *In vitro*, PEEK-PITAc modulates macrophage behavior toward a more pro-regenerative phenotype and establishes an immune microenvironment favorable for osteogenesis and angiogenesis. The modified surface also directly supports the osteogenic and angiogenic functions of relevant cells. Mechanistically, these effects are associated with autophagy-related changes and reduced PI3K-AKT pathway activation. In a rat femoral implantation model, PEEK-PITAc mediates a more favorable local immune response, promotes peri-implant bone formation and improves osseointegration. Overall, this study shows that PITAc functionalization is a simple and effective strategy for endowing bioinert PEEK with enhanced osteoimmunomodulatory and bone-regenerative properties.

## Graphical abstract

**Graphical Abstract:**
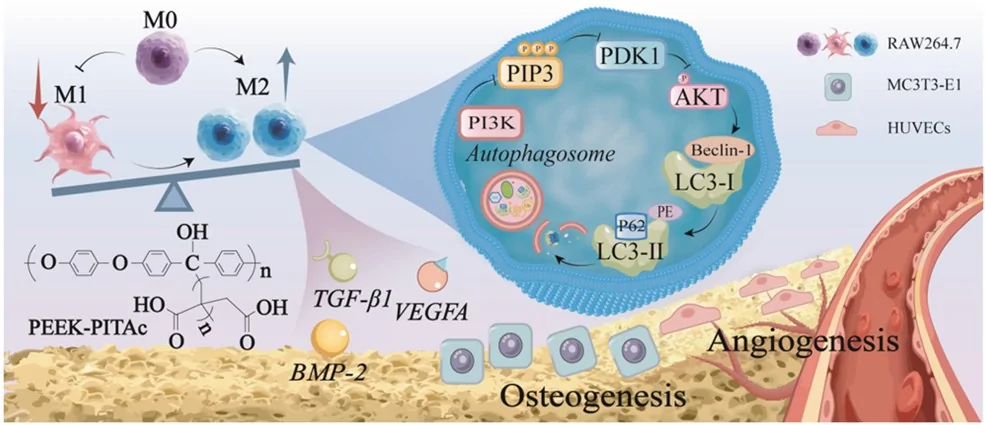

## Introduction

Bone defects are common in clinical orthopedics and arise from trauma, tumors, infection and congenital malformations. As populations age and accidental trauma remains prevalent, the clinical demand for effective bone repair continues to increase [1]. Current treatment still relies largely on metallic implants and fixation devices [2]. Titanium-based implants provide high mechanical strength and good clinical reliability, but their elastic modulus mismatch with native bone tissue can induce stress shielding, peri-implant bone loss and less favorable long-term fixation in some cases [3]. These limitations have driven continued interest in alternative materials for bone regeneration.

Among currently available biomaterials, polyetheretherketone (PEEK) has attracted considerable attention because of its excellent chemical stability, favorable mechanical properties, radiolucency and biocompatibility [4]. In particular, its elastic modulus is closer to that of cortical bone than that of metallic implants, which helps reduce stress shielding and lowers the risk of implant loosening [5]. However, pristine PEEK is bioinert and exhibits limited osteoconductivity, resulting in insufficient bone bonding and poor osseointegration. In addition, unmodified PEEK lacks active regulatory effects on the peri-implant immune microenvironment, which may restrict coordinated osteogenesis and tissue integration [6–8]. Existing surface modification strategies for PEEK, including inorganic coatings, micro- and nanostructure construction, bioactive ion incorporation and biomolecule grafting, have primarily aimed to improve osteogenic activity [9–11]. Although these approaches have achieved partial success, many focus predominantly on direct stimulation of osteoblast-related responses and pay less attention to the host immune reaction that critically influences implant outcomes [12].

With the development of osteoimmunology, it has become increasingly clear that bone regeneration is not determined solely by the osteogenic properties of a material, but also by its ability to regulate the immune environment after implantation [13]. Macrophages are central to this process because of their marked phenotypic plasticity and their role in coordinating inflammation, angiogenesis and bone formation [14]. Classically activated M1 macrophages produce pro-inflammatory mediators such as tumor necrosis factor-α (TNF-α), interleukin-1β (IL-1β) and IL-6, and persistent M1 dominance can lead to chronic inflammation and fibrous encapsulation, thereby impairing bone healing. In contrast, alternatively activated M2 macrophages secrete anti-inflammatory and pro-regenerative factors, including IL-10 and transforming growth factor-β1, which support tissue repair, angiogenesis and osteogenesis [15–17]. Autophagy is closely linked to this immunoregulatory process. By removing damaged organelles and intracellular waste, autophagy helps maintain cellular homeostasis and influences macrophage phenotype and cytokine production [18]. Previous studies have shown that autophagy activation can favor M2 polarization and support osteoblast proliferation and mineralization, whereas impaired autophagy aggravates inflammation and compromises bone regeneration [19, 20]. The PI3K-AKT pathway is one of the major upstream regulators of autophagy and has therefore emerged as a relevant target in the design of osteoimmunomodulatory biomaterials [21].

Itaconic acid (ITAc) is an endogenous immunometabolite produced predominantly by activated macrophages from cis-aconitate during inflammatory stimulation. Structurally, ITAc contains one carbon–carbon double bond and two carboxyl groups. These carboxyl groups can increase surface hydrophilicity, which is generally favorable for protein adsorption and cell attachment [22]. The vinyl group also enables efficient graft polymerization under suitable initiation conditions, making ITAc an attractive candidate for stable surface functionalization [23]. Beyond these chemical features, ITAc has received increasing attention because of its biological activity. It can regulate macrophage metabolism, suppress excessive inflammatory responses and has been associated with promotion of a reparative macrophage phenotype [24]. ITAc has also been linked to autophagy-related signaling, including modulation of the PI3K-AKT axis, suggesting that it may help couple immune regulation with osteogenic repair [25, 26]. To date, however, studies on ITAc have mainly focused on drug development and carrier-based delivery systems, while its direct use in biomaterial surface engineering remains limited [27, 28]. Moreover, whether polyitaconic acid (PITAc) immobilized on a material surface can retain the beneficial bioactivity associated with ITAc has not been well defined.

PEEK contains benzophenone-like moieties in its molecular backbone, which can generate semiquinone radicals under ultraviolet irradiation [29–32]. Taking advantage of this property, we used a one-step UV-initiated graft polymerization strategy to immobilize ITAc on the PEEK surface and generate PITAc-functionalized PEEK (PEEK-PITAc, Figure 1A). Compared with approaches involving specialized deposition equipment or multistep coating assembly [9–12], this process uses photoactive moieties intrinsic to the PEEK backbone and does not require an additional initiator. Its practical advantages therefore lie in process simplicity and stable covalent surface functionalization, while the resulting PITAc layer provides integrated immunomodulatory, osteogenic and angiogenic functions. In this study, we explored PITAc-mediated surface functionalization on a PEEK substrate for bone-regenerative applications. We hypothesized that PEEK-PITAc would regulate the peri-implant immune microenvironment in association with reduced PI3K-AKT activation and autophagy-related changes, thereby favoring macrophage polarization toward a pro-regenerative phenotype and establishing conditions that support angiogenesis and osteogenesis. We further investigated whether this immunoregulatory effect, together with the direct bioactivity of the modified surface, could enhance new bone formation and osseointegration *in vivo* (Figure 1B).

**Figure 1:**
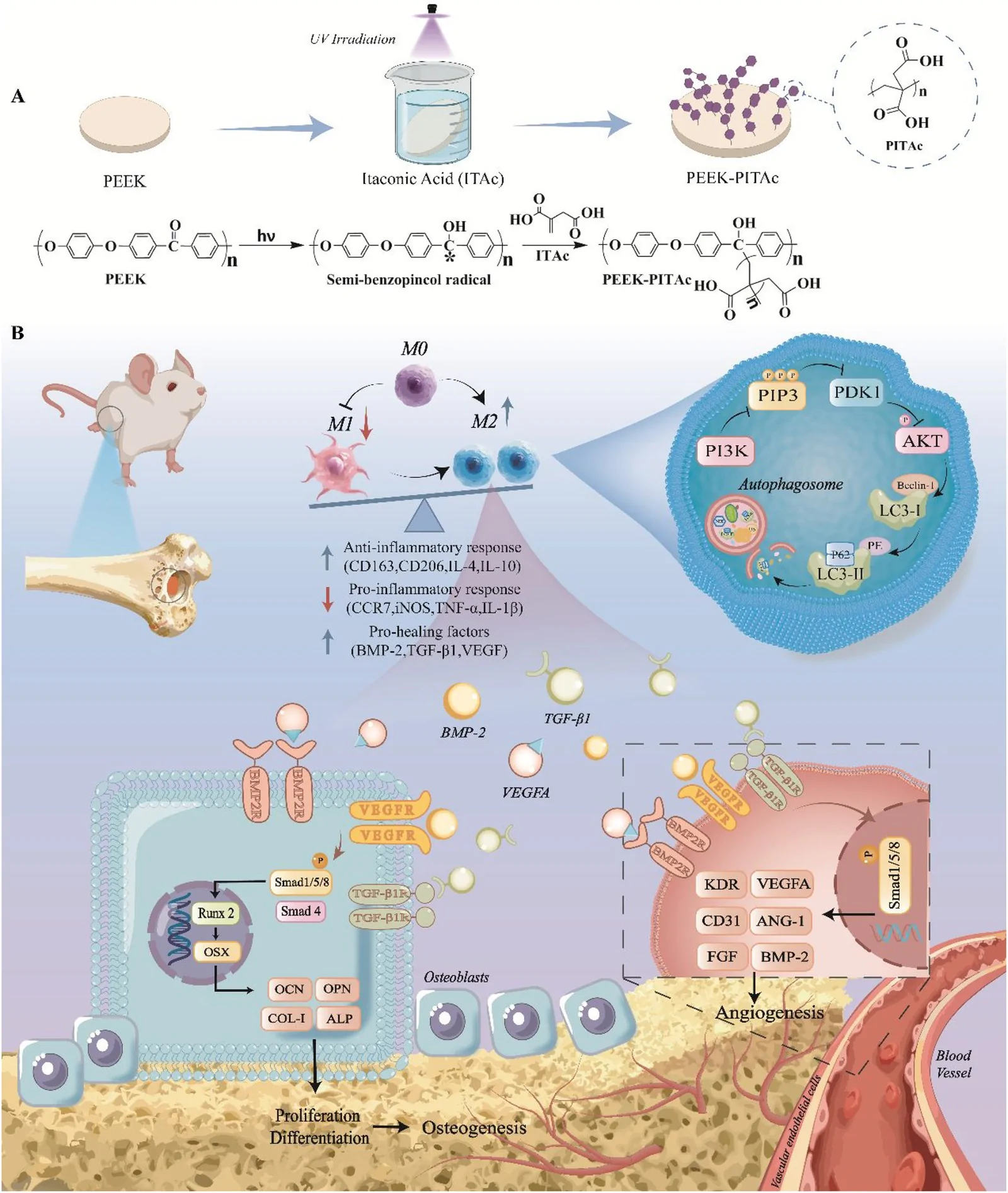
Schematic illustration of PEEK-PITAc preparation and its pro-regenerative mechanism. (**A**) Preparation of PEEK-PITAc. (**B**) activates autophagy through the PI3K-AKT signaling pathway, establishes a pro-regenerative osteoimmune microenvironment, promotes angiogenesis and osteogenesis and ultimately enhances new bone formation and osseointegration *in vivo*.

## Materials and methods

The detailed process of the preparation and characterization of PEEK-PITAc, and *in vitro* and *in vivo* biological experimental procedures are described in Supplementary Information.

## Results and discussion

### Surface characterization

We prepared PITAc-functionalized PEEK by UV-initiated graft polymerization and characterized the surface morphology using SEM and AFM (Figure 2A and B). Both pristine PEEK and PEEK-PITAc surfaces presented flat and smooth. AFM analysis revealed no significant morphological differences between the two groups, with comparable surface roughness values (Figure 2B). This observation can be attributed to the uniform and thin-layer grafting of PITAc onto the PEEK surface.

**Figure 2:**
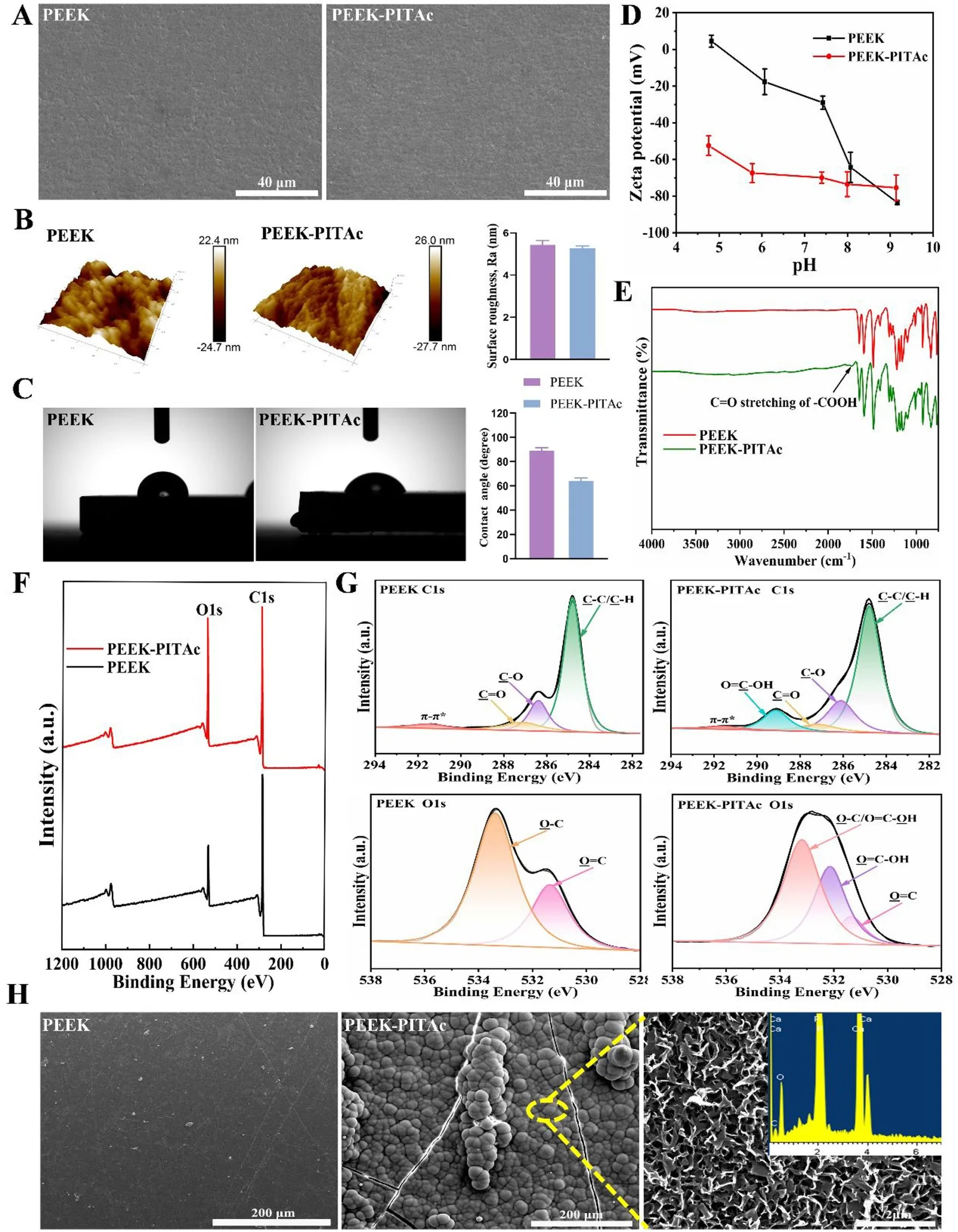
Surface characterization of pristine PEEK and PEEK-PITAc. (**A**) FE-SEM images. (**B**) AFM images and surface roughness determined by AFM (*n* = 3 per group). (**C**) Representative water droplet profiles and water contact angles (*n* = 3 per group). (**D**) Zeta potential values (*n* = 3 per group). (**E**) FTIR spectra. (**F**) XPS survey spectra. (**G**) High-resolution XPS C1s and O1s spectra. (**H**) Surface morphology after 30 days immersion in SBF and corresponding EDS spectra. *P < 0.05 compared to PEEK.

Surface hydrophilicity was evaluated using static water contact angle. Pristine PEEK showed a contact angle of 89.58 ± 1.69°, whereas that of PEEK-PITAc was 64.75 ± 1.47° (Figure 2C). This lower contact angle indicates markedly enhanced hydrophilicity after PITAc grafting, which is expected from the abundant hydrophilic –COOH groups in the PITAc. Better hydrophilicity facilitates cell adhesion, spreading and proliferation, and benefits subsequent immune regulation, angiogenesis, osteogenesis and bone regeneration [33]. Surface zeta potential is an important determinant of biological responses [34]. Both samples became more negative as pH increased (Figure 2D). At pH 7.4, PEEK-PITAc showed a significantly lower zeta potential than pristine PEEK, which is attributable to ionized surface –COOH groups.

FTIR analysis identified the characteristic C = O stretching vibration of –COOH groups at 1730 cm^−1^, confirming successful PITAc grafting onto the PEEK surface (Figure 2E). XPS further showed stronger O1s intensity and a higher O1s/C1s atomic ratio on PEEK-PITAc than on pristine PEEK (Figure 2F and Supplementary Table S2), consistent with the oxygen-rich (44.44%) structure of PITAc. In the high-resolution spectra, the –COOH peak at 289.1 eV in the C1s spectrum provides additional evidence of grafting (Figure 2G). The remaining C = O peak at 287.2 eV suggests that the PITAc layer is relatively thin, with a thickness lower than or comparable to the effective XPS probing depth. After UV irradiation, benzophenone-like groups in PEEK generate semiquinone radicals, which reduce the C = O contribution and increase the C–O contribution at 286.2 eV. In the O1s spectrum (Figure 2G), both intrinsic O–C groups in PEEK and newly formed O–C groups contribute to the 533.3 eV peak, so this peak is larger than the 532.1 eV peak. The fitted peak fractions are summarized in Supplementary Table S3.

To evaluate bioactivity, we immersed pristine PEEK and PEEK-PITAc in SBF. After 30 days, pristine PEEK remained smooth and showed no obvious deposition, consistent with its inert surface (Figure 2H). In contrast, PEEK-PITAc was uniformly covered by granular deposits composed of nanoscale flaky crystals, similar to our previous observations [35]. EDS analysis revealed a Ca/P atomic ratio of 1.63 (Supplementary Table S4), close to stoichiometric hydroxyapatite at 1.67, indicating that these deposits were bone-like apatite [36]. These results show that PITAc functionalization substantially improves the bioactivity of PEEK and establishes a favorable surface for subsequent bone regeneration and osseointegration.

### Effects of PEEK-PITAc on the immune responses of RAW264.7 cells and potential molecular mechanisms

Following orthopedic implantation, immune cells rapidly accumulate around orthopedic implants and remodel the peri-implant milieu through secreted signals, thereby shaping later bone regeneration [37]. We therefore cultured RAW264.7 macrophages on the material surfaces to examine how PEEK-PITAc influences early immune responses.

SEM revealed clear differences in cell morphology (Figure 3A). After 4 h, macrophages on the PEEK surface appeared elliptical, whereas cells on PEEK-PITAc remained rounded. After 24 h, cells on PEEK spread extensively and extended pseudopodia, while those on PEEK-PITAc still showed a smaller adhesion area. This morphology is consistent with previous reports that rounded macrophages on biomaterials more readily adopt an M2-like phenotype [38]. Live/Dead staining at Day 5 showed intense green fluorescence and minimal red fluorescence on both surfaces (Figure 3B), and CCK-8 assays confirmed sustained proliferation over 1, 3 and 5 days (Figure 3C). Both materials therefore supported macrophage viability.

**Figure 3:**
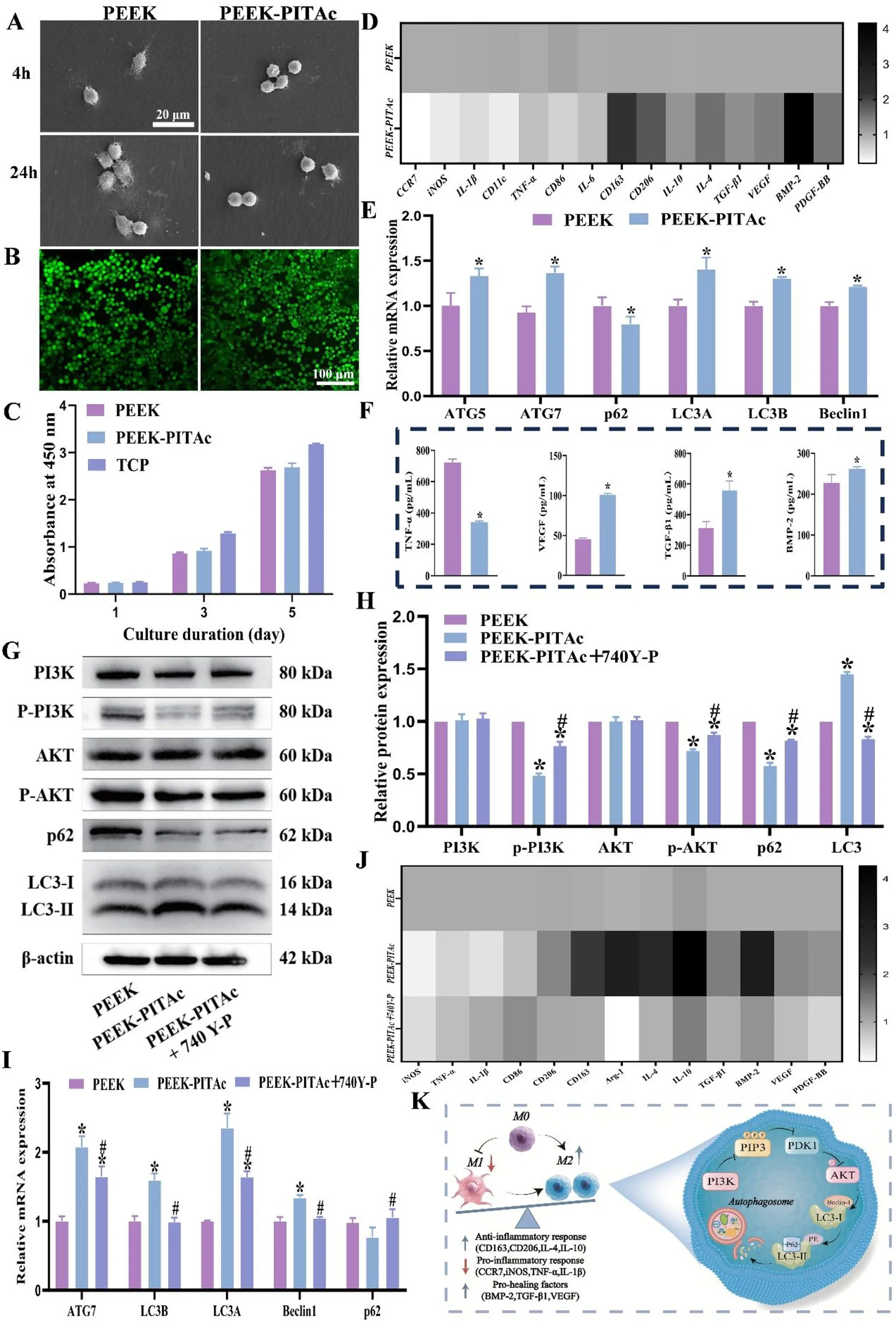
PEEK-PITAc Modulates the immune responses of RAW264.7 cells *in vitro* and its potential molecular mechanisms. (**A**) Cells morphologies. (**B**) Live/dead staining at 5 days of incubation. (**C**) CCK-8 assay at 1, 3 and 5 days post-seeding (*n* = 3 per group). TCP denotes tissue-culture polystyrene and represents cells cultured without material specimens. (**D**) Heatmap summarizing the expression of M1-associated genes (CCR7, iNOS, IL-1β, CD11c, TNF-α, CD86 and IL-6), M2-associated genes (CD163, CD206, IL-10, and IL-4), and osteogenesis- and angiogenesis-related genes (TGF-β1, VEGF, BMP-2 and PDGF-BB). (**E**) RT-qPCR detection of the expression of autophagy-associated genes (ATG5, ATG7, P62, LC3A, LC3B and Beclin1) (*n* = 3 per group). (**F**) ELISA analysis of TNF-α, VEGF, TGF-β1 and BMP-2 (*n* = 3 per group). (**G**) Protein band display and (**H**) relative gray value analysis of PI3K, p-PI3K, AKT, p-AKT, p62, LC3-I and LC3-II in different samples with adding PI3K agonist 740 Y-P. (**I**) Relative mRNA expression of autophagy-associated genes (ATG7, LC3A, p62, Beclin1 and LC3B) in different samples with adding PI3K agonist 740 Y-P (*n* = 3 per group). (**J**) Heatmap summarizing the expression of M1-associated genes (iNOS, IL-1β, TNF-α and CD86), M2-associated genes (CD163, CD206, arg-1, IL-10 and IL-4) and osteogenesis- and angiogenesis-related genes (TGF-β1, VEGF, BMP-2 and PDGF-BB) in different samples with adding PI3K agonist 740 Y-P. (**K**) Schematic representation of the potential molecular mechanisms by which PEEK‑PITAc regulates the immune microenvironment. *P < 0.05 compared to PEEK, ^#^*P *< 0.05 compared to PEEK-PITAc.

To elucidate the modulatory effect of PEEK-PITAc on RAW264.7 polarization, we quantified the expression of M1-associated genes (IL-1β, TNF-α, IL-6, CCR7, iNOS, CD11c) and M2-associated genes (IL-10, IL-4, CD163, CD206) after 3 days of culture through RT-qPCR analysis. PEEK-PITAc downregulated all measured M1 markers and upregulated all measured M2 markers relative to pristine PEEK (Figure 3D, Supplementary Figure S2A and B). These data show that PEEK-PITAc suppresses M1 polarization and favors the M2 phenotype. This shift may arise from several surface features. The hydrophilic PITAc layer likely improves adsorption of anti-inflammatory matrix proteins such as laminin. Surface –COOH groups can also interact with receptors such as integrins and trigger anti-inflammatory signaling [39, 40]. In addition, the lower zeta potential of PEEK-PITAc may contribute to M2 polarization through inhibition of the PI3K-AKT-mTOR axis [41].

ITAc and its derivatives are also linked to autophagy regulation through PI3K-AKT signaling. Because autophagy maintains intracellular homeostasis and restrains inflammasome activation [42, 43], we measured key autophagy genes. PEEK-PITAc increased ATG5, ATG7, Beclin1, LC3A and LC3B and reduced p62 relative to pristine PEEK (Figure 3E). This pattern is consistent with increased autophagy and/or autophagosome turnover, and suggests that autophagy-related processes may contribute to the immunoregulatory effect of PEEK-PITAc.

Immune regulation is closely linked to bone regeneration because macrophages secrete factors that control osteogenic and angiogenic cell behavior [44]. RAW264.7 cells cultured on PEEK-PITAc expressed higher levels of TGF-β1, VEGF, BMP-2 and PDGF-BB than cells cultured on pristine PEEK (Figure 3D and Supplementary Figure S2C). ELISA confirmed lower TNF-α secretion and higher release of osteogenic and angiogenic factors in the PEEK-PITAc group (Figure 3F). These results indicate that PEEK-PITAc not only dampens inflammation but also programs macrophages to support bone regeneration.

The PI3K-AKT pathway is a central regulator of autophagy in macrophages [45]. To examine whether the immunomodulatory effects of PEEK-PITAc were associated with changes in this pathway and autophagy-related activity, we measured pathway proteins, autophagy markers and immunoregulatory genes by western blot and RT-qPCR. Total PI3K and AKT levels were similar among groups, indicating that PEEK-PITAc did not alter basal expression of these proteins (Figure 3G and H). In contrast, phosphorylated PI3K and AKT were lower in the PEEK-PITAc group than in the PEEK group and were restored in the PEEK-PITAc + 740Y-P group. This shows that PEEK-PITAc mainly affects pathway activation rather than total protein abundance. Changes in autophagy markers matched this pattern. In the PEEK-PITAc group, p62 protein was reduced and LC3-II was increased, whereas LC3-I changed little (Figure 3G and H). These results are consistent with increased autophagy and/or autophagosome turnover. Both effects were reversed by 740Y-P treatment, supporting an association between PI3K-AKT signaling and these autophagy-related changes. RT-qPCR showed the same trend. Autophagy-associated genes, including ATG7, LC3B, LC3A and Beclin1, were upregulated in the PEEK-PITAc group and downregulated after 740Y-P treatment, whereas p62 showed the opposite pattern (Figure 3I).

Immune phenotype markers also depended on this pathway. Compared with pristine PEEK, PEEK-PITAc reduced M1-associated genes, including iNOS, TNF-α, IL-1β and CD86, and increased M2-associated genes including CD206, CD163, Arg-1, IL-4 and IL-10 (Figure 3J, Supplementary Figure S3A and B). These changes were weakened or reversed by 740Y-P. Osteogenic and angiogenic genes followed the same trend as the M2 markers (Figure 3J and Supplementary Figure S3C). Together, these results show that PEEK-PITAc is associated with reduced PI3K-AKT phosphorylation and concurrent autophagy-related changes. This shift restrains pro-inflammatory polarization, promotes the M2 phenotype and helps build a regenerative osteoimmune microenvironment for bone repair (Figure 3K). Autophagy-specific inhibition or genetic silencing will be required to determine whether autophagy is essential for these responses and whether macrophage phenotypic regulation directly mediates the downstream factor profile.

### Effect of immune microenvironment on osteogenesis and angiogenesis

Bone regeneration depends on coordinated osteogenesis and angiogenesis. Blood vessels deliver oxygen and nutrients and remove waste, making angiogenesis essential for bone repair [46]. Because early immune responses affect neovascularization and bone formation, we used an indirect co-culture system to test how the PEEK-PITAc-conditioned immune microenvironment influences osteogenic differentiation of MC3T3-E1 pre-osteoblasts (Figure 4A) and HUVEC function (Figure 4F).

**Figure 4:**
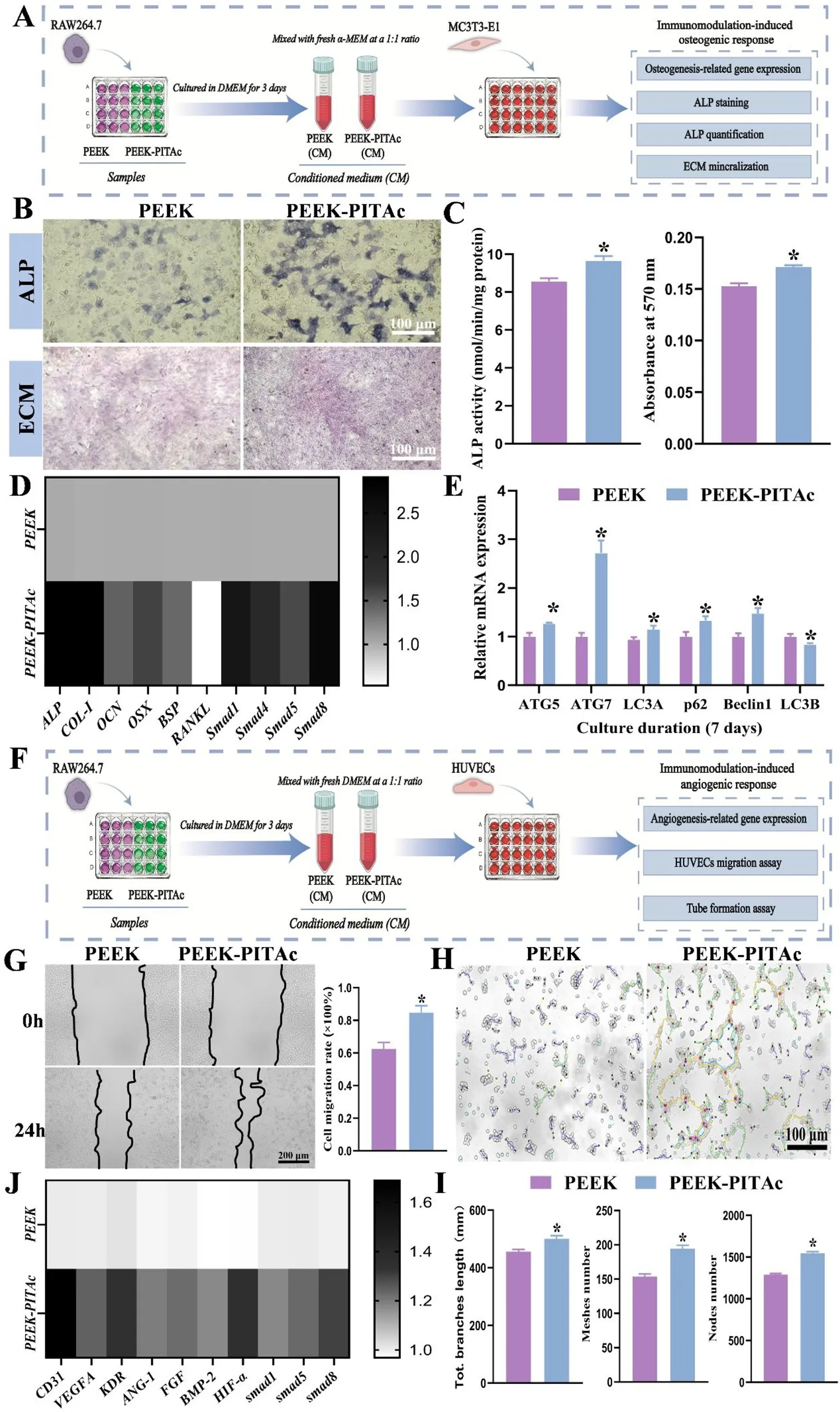
Effects of the SMCM on osteogenesis of MC3T3-E1 pre-osteoblasts and angiogenesis of HUVECs. (**A**) The experimental design for evaluating osteogenesis via SMCM. (**B**) Qualitative staining images of ALP and ECM mineralization. (**C**) Quantitative ALP activity and ECM mineralization (*n* = 3 per group). (**D**) Heatmap summarizing the expression of osteogenesis-related genes (ALP, COL-I, OCN, OSX, BSP and RANKL) and the expression of Smad1, smad 4, Smad5 and smad 8 (*n* = 3 per group). (**E**) RT-qPCR detection of the expression of autophagy-associated genes (ATG5, ATG7, LC3A, P62, Beclin1 and LC3B). (**F**) The experimental design for evaluating angiogenesis via SMCM. (**G**) Representative migration images of HUVECs after incubation of 0 and 24 h and the migration ratio (*n* = 3 per group). (**H**) Representative vascular network images and quantitative data (**I**) for total branching length, mesh number and node number following 24 h culture on Matrigel (*n* = 3 per group). (**J**) Heatmap summarizing the expression of angiogenesis-related genes (CD31, VEGFA, KDR, ANG-1, FGF, BMP-2 and HIF-α) and the expression of Smad1, Smad5 and Smad8 expression. *P < 0.05 compared to PEEK.

ALP activity and ECM mineralization reflect early and late osteogenic differentiation. At 7 and 14 days, MC3T3-E1 cells treated with sample/macrophage-conditioned medium (SMCM) from PEEK-PITAc showed stronger ALP staining and more extensive mineralized matrix than cells treated with SMCM from PEEK (Figure 4B). Quantification confirmed these differences (Figure 4C). RT-qPCR further showed significantly higher expression of osteogenic-related genes, including ALP, COL-I, OCN, OSX and BSP, and lower expression of osteoclastogenesis-related gene RANKL in the PEEK-PITAc SMCM group (Figure 4D and Supplementary Figure S4A). We then examined possible mechanisms. RAW264.7 cells on PEEK-PITAc secreted high levels of TGF-β1, BMP-2, VEGF and PDGF-BB, all of which are known to support osteogenesis. TGF-β1 and BMP-2 activate Smad signaling in pre-osteoblasts [47], and SMCM from PEEK-PITAc increased Smad1, Smad4, Smad5 and Smad8 expression compared with SMCM from PEEK (Figure 4D and Supplementary Figure S4B). VEGF can cooperate with BMP-2 to enhance osteoblast differentiation [48], while PDGF-BB promotes osteoblast proliferation and differentiation through Src/JAK2 signaling [49]. Autophagy is also important for osteoblast homeostasis and differentiation. MC3T3-E1 cells exposed to PEEK-PITAc SMCM showed higher expression of autophagy-related genes than cells exposed to PEEK SMCM (Figure 4E). These results suggest that PEEK-PITAc creates a pro-osteogenic immune microenvironment by coupling paracrine osteogenic signals with enhanced autophagy.

It was also found that the PEEK-PITAc SMCM exhibited significant effects on angiogenesis. Endothelial cell migration is an essential prerequisite for neovascularization. A scratch assay showed faster migration of HUVECs treated with SMCM from PEEK-PITAc than with SMCM from PEEK at 24 h (Figure 4G). In the Matrigel assay, SMCM from PEEK-PITAc supported a denser and more interconnected vascular network, whereas SMCM from PEEK yielded only sparse and short tube-like structures (Figure 4H). Quantification of total branch length, mesh number and node number confirmed the stronger angiogenic response in the PEEK-PITAc group (Figure 4I). PEEK-PITAc SMCM also increased expression of endothelial maturation marker CD31, core VEGF signaling pathway genes (VEGFA, KDR), pro-vascular stabilization/branching genes (ANG-1, FGF) and dual-functional osteogenic-angiogenic genes (BMP-2, HIF-α) in HUVECs (Figure 4J and Supplementary Figure S5A). These effects are consistent with the macrophage secretory profile induced by PEEK-PITAc. RAW264.7 cells on PEEK-PITAc produced more VEGF, TGF-β1 and BMP-2, all of which can support vascular growth. VEGF activates canonical VEGF/VEGFR signaling to drive endothelial proliferation and migration, processes that also facilitate tube assembly and maturation [50]. TGF-β1 and BMP-2 can further activate Smad signaling in HUVECs and enhance transcription of pro-angiogenic genes. RT-qPCR analysis of the Smad expression demonstrated significantly higher Smad1, Smad5 and Smad8 expression in HUVECs treated with SMCM from PEEK-PITAc than with SMCM from PEEK (Figure 4J and Supplementary Figure S5B). Thus, PEEK-PITAc creates a pro-angiogenic immune microenvironment through coordinated VEGF/VEGFR and TGF-β1/BMP-2-Smad signaling.

Taken together, these findings establish a mechanistic link between the PI3K-AKT-associated autophagy response in macrophages and the subsequent osteogenic and angiogenic effects. PEEK-PITAc reduced PI3K and AKT phosphorylation and induced concurrent changes in autophagy-related markers, which were accompanied by suppression of the pro-inflammatory macrophage phenotype and promotion of a pro-regenerative phenotype. These macrophages exhibited increased expression and secretion of VEGF, TGF-β1, BMP-2 and PDGF-BB, thereby generating a paracrine microenvironment favorable for vascularization and bone formation. VEGF-related signaling is consistent with the enhanced migration and tube formation of HUVECs and the increased expression of VEGFA, KDR, CD31, ANG-1 and FGF. Meanwhile, BMP-2- and TGF-β1-associated signaling, together with increased Smad-related gene expression, is consistent with the enhanced osteogenic differentiation, extracellular matrix production and mineralization of MC3T3-E1 cells. Thus, PEEK-PITAc couples macrophage PI3K-AKT/autophagy regulation with the secretion of regenerative mediators, which subsequently coordinate angiogenic and osteogenic responses.

### Evaluation of osteoimmunomodulatory capacities of the samples in direct co-culture

Macrophage-osteoblast crosstalk is central to bone repair and occurs through both paracrine signaling and direct cell–cell contact [51]. Our indirect co-culture experiments showed that the PEEK-PITAc-conditioned immune environment promotes osteogenesis and angiogenesis via macrophage secretions. To test whether this effect persists under more physiologically relevant contact conditions, we established a direct RAW264.7-MC3T3-E1 co-culture system on the material surfaces (Figure 5A).

**Figure 5:**
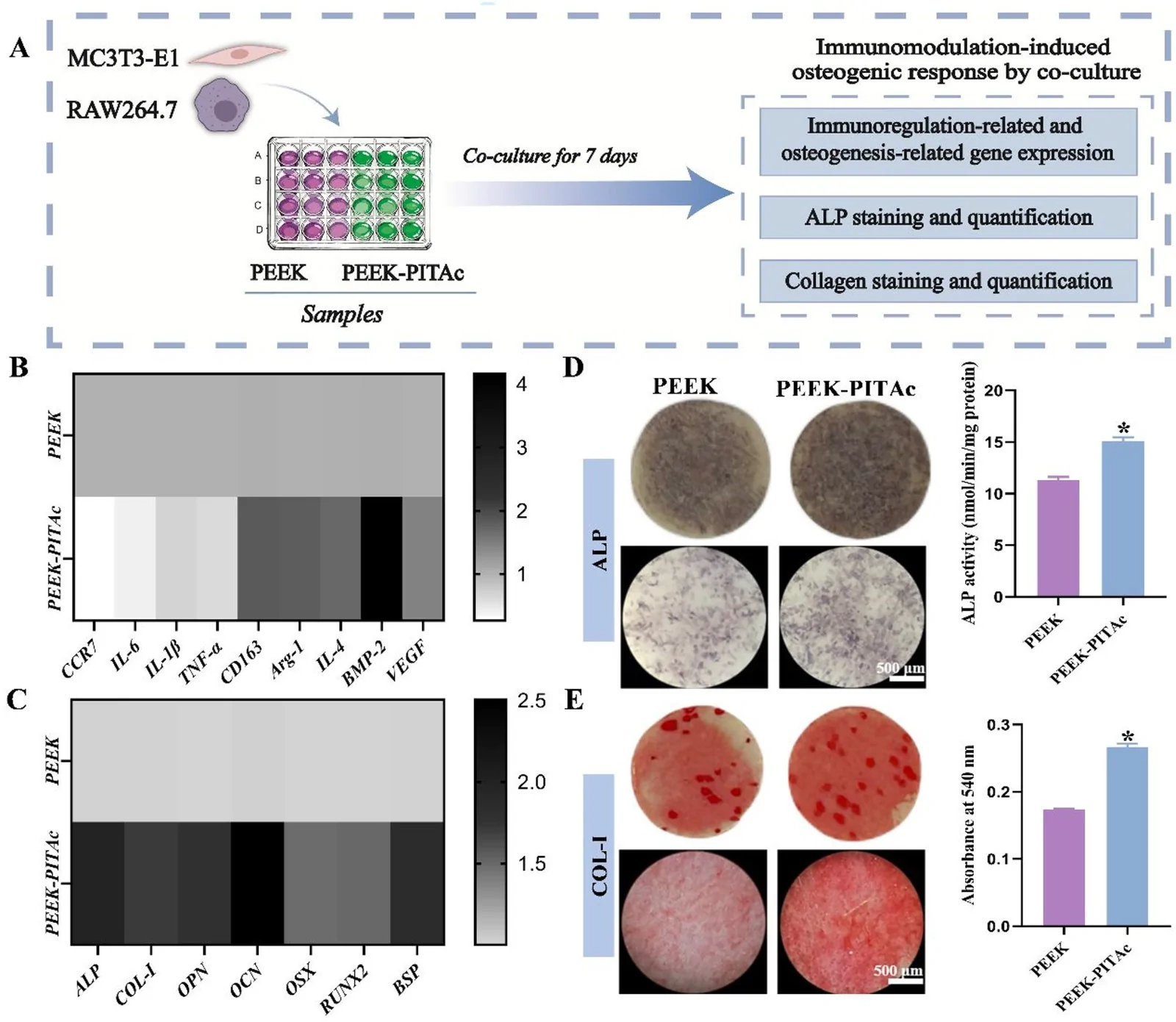
Assessment of the osteoimmunomodulatory capacity of various samples at 7 days of direct co-culture. (**A**) Schematic illustration of the direct co-culture experimental design. (**B**) Heatmap summarizing the expression of M1-associated genes (CCR7, IL-6, IL-1β and TNF-α), M2-associated genes (CD163, Arg-1 and IL-4) and osteogenesis- and angiogenesis-related genes (BMP-2 and VEGF). (**C**) Heatmap summarizing the expression of osteogenesis-related gene. (**D**) ALP activity qualitative staining images and quantitative results (*n* = 3 per group). (**E**) COL-I secretion qualitative staining images and quantitative results (*n* = 3 per group). *P < 0.05 compared to PEEK.

After 7 days of RAW264.7-MC3T3-E1 co-culture on PEEK and PEEK-PITAc, macrophage polarization and the osteogenic activity of MC3T3-E1 cells were assessed. PEEK-PITAc markedly reduced the expression of M1-associated genes, including CCR7, IL-6, IL-1β and TNF-α, while upregulating the expression of M2-associated genes, including CD163, Arg-1 and IL-4, together with osteogenesis- and angiogenesis-related genes, including BMP-2 and VEGF (Figure 5B, Supplementary Figure S6A and B). Consistently, the expression of osteogenesis-related genes, including ALP, COL-I, OPN, OCN, OSX, Runx2 and BSP, was significantly higher in the PEEK-PITAc group than in the PEEK group (Figure 5C and Supplementary Figure S6C). Staining results supported this trend. ALP staining and activity quantification indicated markedly increased ALP activity in the PEEK-PITAc group, indicating enhanced early osteogenic differentiation of MC3T3-E1 cells (Figure 5D). Similarly, PEEK-PITAc showed stronger sirius red staining than the PEEK, indicating enhanced collagen deposition (Figure 5E). These findings indicate that PEEK-PITAc promotes osteogenesis by sustaining pro-regenerative macrophage-preosteoblast crosstalk through both paracrine signaling and direct cell–cell contact. Future studies incorporating direct macrophage-HUVEC co-culture will further clarify the corresponding angiogenic crosstalk.

### Direct osteogenic functionality of MC3T3-E1 pre-osteoblasts and angiogenic functionality of HUVECs on different samples

Angiogenesis and osteogenesis occur concomitantly during bone formation. Besides the indirect osteogenic and angiogenic effects mediated by the immune microenvironment, implant surface properties can also directly regulate osteoblast and endothelial behavior [52]. We therefore cultured MC3T3-E1 pre-osteoblasts and HUVECs directly on PEEK and PEEK-PITAc to assess whether PITAc functionalization confers an intrinsic osteogenic effect and pro-angiogenic cues.

SEM showed more favorable early adhesion on PEEK-PITAc (Figure 6A). After 4 h, cells on PEEK were mainly round, spindle-shaped or only partly spread, whereas cells on PEEK-PITAc appeared flatter and more firmly attached. After 24 h, spreading increased on both surfaces, but near-confluent cell coverage was observed only on PEEK-PITAc. Live/dead fluorescence staining at Day 5 (Figure 6B) revealed that cells on both materials displayed intense green fluorescence with negligible red fluorescence. CCK-8 proliferation assay results (Figure 6C) indicated that both PEEK and PEEK-PITAc efficiently promoted MC3T3-E1 proliferation at all time points, with the cell number in the PEEK-PITAc group being significantly higher than that in the PEEK group on Day 5. Gene expression analysis at 7 and 14 days further showed higher levels of ALP, COL-I, OCN, OSX and BSP on PEEK-PITAc than on PEEK (Figure 6D and Supplementary Figure S7). Qualitative and quantitative assays of ALP activity, collagen secretion and ECM mineralization all showed the same trend, with stronger osteogenic responses on PEEK-PITAc throughout culture (Figure 6E–G). These improvements are consistent with the surface chemistry. The introduced –COOH groups increase hydrophilicity and support cell adhesion, spreading and proliferation [53, 54]. Under physiological conditions, these groups are negatively charged, which can chelate Ca^2+^ and enhance osteogenic cue transduction [55]. Negatively charged surfaces further favor adsorption of adhesive proteins such as fibronectin while reducing nonspecific adsorption of proteins such as albumin [56]. PEEK-PITAc therefore promotes osteogenesis both directly through surface cues and indirectly through osteoimmunomodulation.

**Figure 6:**
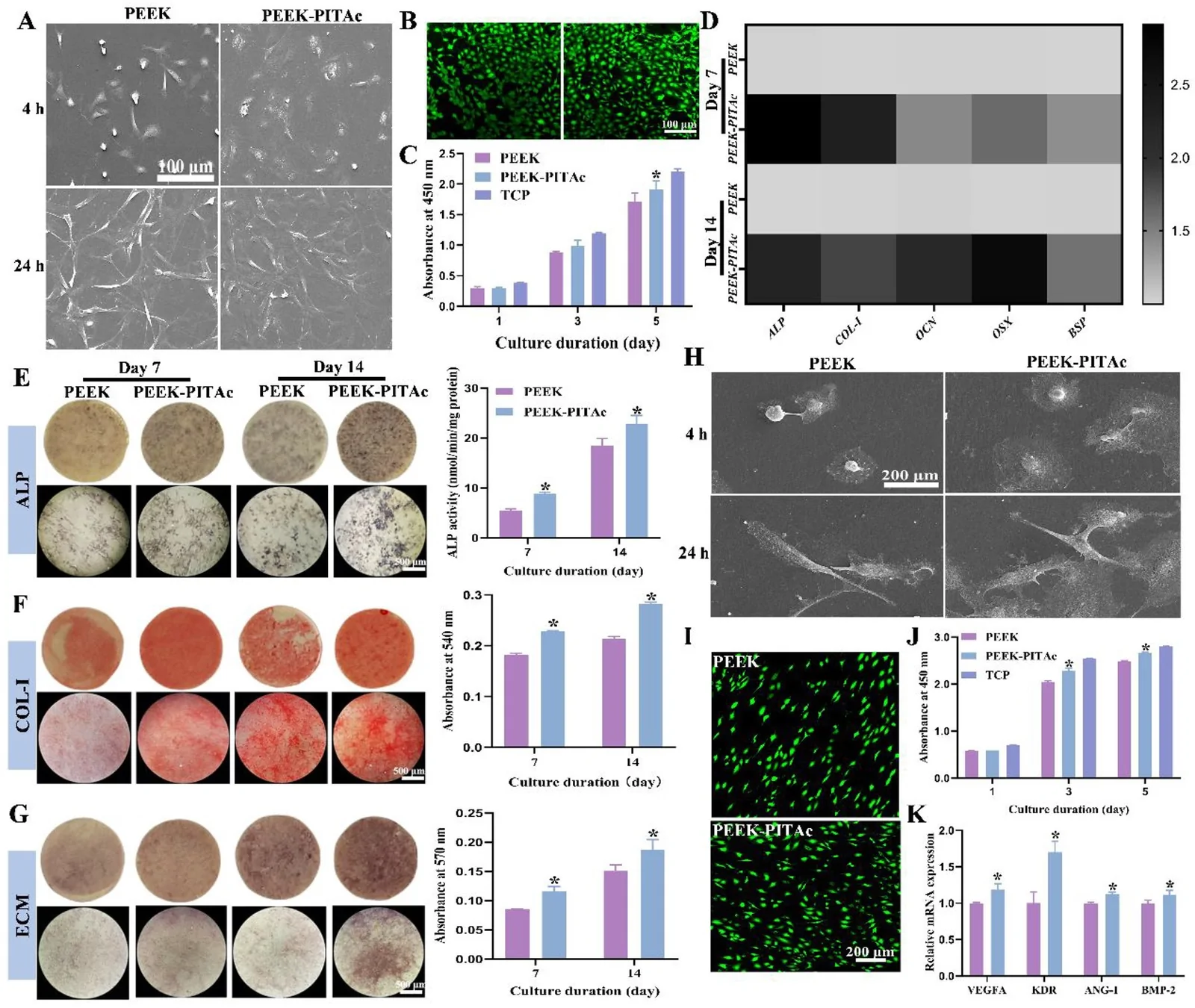
PEEK-PITAc Directly enhances osteogenic functionality of MC3T3-E1 pre-osteoblasts and angiogenic performance of HUVECs *in vitro*. (**A**) MC3T3-E1 cell morphologies at 4 and 24 h post-seeding. (**B**) Live/dead staining of MC3T3-E1 cells at 5 days post-seeding. (**C**) CCK-8 assay at 1, 3 and 5 days post-seeding of MC3T3-E1 cells (*n* = 3 per group). TCP denotes tissue-culture polystyrene and represents cells cultured without material specimens. (**D**) Heatmap summarizing the expression of osteogenesis-related gene at 7 and 14 days post-seeding. (**E**) ALP activity staining images and quantitative results at 7 and 14 days post-seeding (*n* = 3 per group). (**F**) COL-I secretion staining images and quantitative results at 7 and 14 days post-seeding (*n* = 3 per group). (**G**) ECM mineralization staining images and quantitative results at 7 and 14 days post-seeding (*n* = 3 per group). (**H**) HUVECs morphologies at 4 and 24 h post-seeding. (**I**) Live/dead staining of HUVECs at 3 days post-seeding. (**J**) CCK-8 assay at 1, 3 and 5 days post-seeding of HUVECs (*n* = 3 per group). TCP denotes tissue-culture polystyrene and represents cells cultured without material specimens. (**K**) Relative mRNA expression of angiogenesis-related genes (VEGFA, KDR, ANG-1 and BMP-2) at 3 days post-seeding (*n* = 3 per group). *P < 0.05 compared to PEEK.

It was found that PEEK-PITAc also directly regulated angiogenesis. SEM showed that HUVECs adhered and spread more effectively on PEEK-PITAc (Figure 6H). After 4 h, cells on PEEK were mainly spherical or only partly flattened, whereas cells on PEEK-PITAc were already broadly spread. After 24 h, cells on PEEK remained elongated with limited intercellular contacts, while those on PEEK-PITAc formed extensive spreading and stronger cell–cell interactions. Live/Dead staining after 3 days showed high viability on both materials (Figure 6I), and CCK-8 assays confirmed sustained proliferation over 1, 3 and 5 days (Figure 6J). RT-qPCR further revealed higher expression of angiogenic genes including VEGFA, KDR, ANG-1 and BMP-2 on PEEK-PITAc than on PEEK (Figure 6K). These results show that PEEK-PITAc not only promotes angiogenesis indirectly through osteoimmunomodulation but also directly enhances endothelial adhesion, growth and angiogenic gene expression. The combination of these direct and indirect effects would support vascularized bone regeneration. Whether PEEK-PITAc-treated MC3T3-E1 cells secrete angiogenic factors that promote endothelial activity was not examined in this study and warrants further investigation. Together with the conditioned-medium and direct co-culture results, these findings indicate that PEEK-PITAc promotes osteogenic and angiogenic responses through complementary macrophage-mediated immunoregulation and direct cell-material effects.

### Effect of PEEK-PITAc on *in vivo* osseointegration

The *in vitro* data indicated that PEEK-PITAc promoted bone repair through both osteoimmunomodulatory and direct cellular effects. We therefore evaluated its performance in an SD rat femoral defect model to assess its *in vivo* capabilities of osteoimmunomodulation, osteogenesis and osseointegration (Figure 7A).

**Figure 7:**
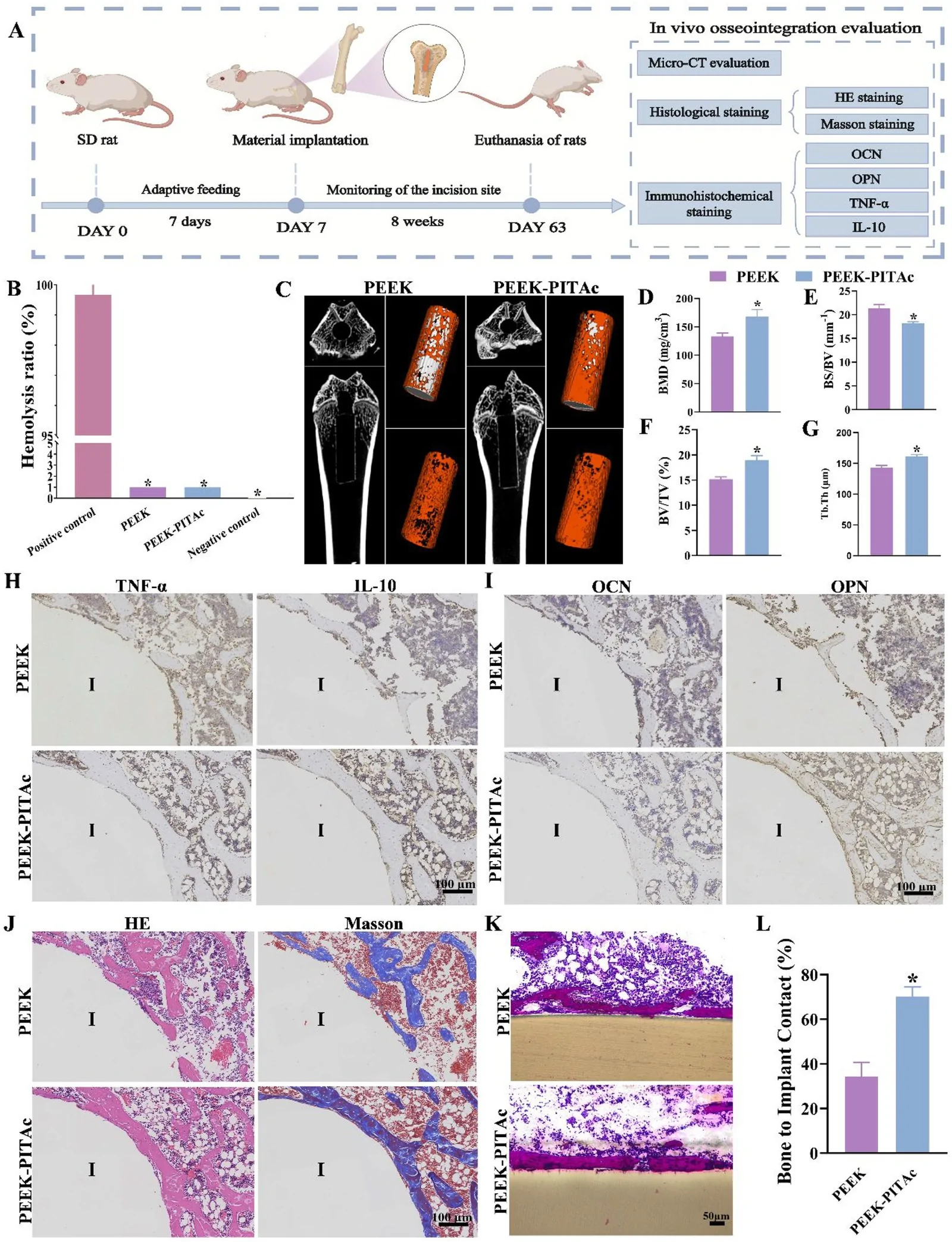
PEEK-PITAc Modulates osseointegration *in vivo*. (**A**) Schematic illustration of the *in vivo* osseointegration evaluation. (**B**) Quantitative results of the *in vitro* hemolysis assay. (**C**) Reconstructed transverse, coronal and 3D micro-CT images. (**D–G**) Quantified micro-CT parameters (BMD, BS/BV, BV/TV, Tb.Th) (*n* = 4 per group). (**H**) Representative immunohistochemical images of TNF-α and IL-10 in the peri-implant tissues. (**I**) Representative immunohistochemical images of OCN and OPN in the peri-implant tissues. (**J**) H&E staining and Masson staining images of the peri-implant tissues. ‘I’ denotes the implant. (**K**) Toluidine blue-acid fuchsin staining images of the peri-implant tissues. (**L**) Quantitative analysis of bone to implant contact percentage. *P < 0.05 compared to PEEK.

Prior to implantation, we conducted hemocompatibility testing on both samples to exclude potential adverse blood-related effects. Both PEEK and PEEK-PITAc exhibited hemolysis rates below 5%, indicating low hemolytic activity under the present test conditions (Figure 7B and Supplementary Figure S8), confirming the non-hemolytic properties and favorable blood compatibility of both materials.

Micro-CT analysis of femurs at 8 weeks post-implantation revealed abundant new bone tissue (red areas) surrounding the PEEK-PITAc implant compared with PEEK control (Figure 7C). Quantitative analysis of bone parameters (Figure 7D–G) showed significantly higher BV/TV, BMD and Tb.Th, and lower BS/BV in the PEEK-PITAc group, indicating that PEEK-PITAc implants induced more abundant new bone formation with higher mineralization levels and a thicker, more stable trabecular bone architecture. Both the quantity and quality of newly formed bone around PEEK-PITAc implants were thus significantly superior to those around PEEK implants.

Histological staining further assessed osseointegration and osteogenesis. Regarding immune responses, the PEEK-PITAc group showed substantially reduced TNF-α expression and enhanced IL-10 expression compared with the PEEK group (Figure 7H), indicating a more favorable local immune microenvironment at 8 weeks. As the present analysis was limited to the terminal 8-week endpoint, future studies will include a 4-week cohort and later time points to characterize the early evolution of peri-implant inflammation and its temporal relationship with subsequent bone formation. Osteogenesis-related proteins OPN and OCN showed substantially higher expression in the PEEK-PITAc group (Figure 7I), confirming its superior osteogenic potential. H&E staining and Masson staining showed limited, discontinuous new bone formation around the PEEK implant, whereas the PEEK-PITAc group exhibited extensive, continuous new bone formation (Figure 7J). Toluidine blue-acid fuchsin staining (Figure 7K) revealed large gaps between newly formed bone tissue and the PEEK implant surface with minimal direct bone-implant contact, indicating poor osseointegration. By contrast, the PEEK-PITAc implant showed continuous, tightly bonded bone tissue formation. Quantitative assessment confirmed a significantly higher bone-implant contact percentage in the PEEK-PITAc group (Figure 7L).

These results are consistent with our *in vitro* findings and confirm that PEEK-PITAc delivers favorable physicochemical signals to orchestrate immune responses and subsequent bone regeneration, ultimately enhancing osseointegration between the implant and newly formed bone tissue. Compared with previously reported PEEK-based materials designed primarily to enhance individual functions such as osteogenesis, angiogenesis, antibacterial activity or immunomodulation [6–11], the distinguishing feature of PEEK-PITAc lies in the integration of multiple biologically relevant activities within a single covalently grafted interface. PEEK-PITAc not only directly supports osteogenic and angiogenic cell responses but also modulates macrophage behavior to establish a pro-regenerative immune microenvironment, thereby coordinating immune regulation, vascularization, bone formation and osseointegration.

## Conclusion

A multifunctional PITAc-functionalized PEEK is fabricated via UV-induced graft polymerization. PITAc grafting improves surface hydrophilicity, reduces zeta potential and significantly enhances bone-like apatite-forming capacity. *In vitro,* PEEK-PITAc modulates macrophage polarization toward the anti-inflammatory M2 phenotype in association with reduced PI3K-AKT phosphorylation and autophagy-related changes. This immune modulation created a microenvironment that favored the angiogenic differentiation of HUVECs and the osteogenic differentiation of MC3T3-E1 pre-osteoblasts. The modified surface also directly enhanced the angiogenic and osteogenic activities of both cell types. In an SD rat femoral defect model, these combined effects translated into stronger bone formation and improved osseointegration *in vivo*. PEEK-PITAc therefore provides a simple fabrication strategy to construct an immunomodulatory and osteogenic PEEK orthopedic implant, and offers new insights into the design of advanced immunomodulatory and bone regenerative materials.

## Supplementary Material

rbag169_Supplementary_Data
