# Supplementary material for: UV-grafted polyitaconic acid functionalization of PEEK promotes osteoimmunomodulation and osseointegration with PI3K-AKT-associated autophagy regulation
Source: Regen Biomater. 2026 Aug 5;13:rbag169. doi: 10.1093/rb/rbag169 (PMC13626135; doi:10.1093/rb/rbag169)
Supplement: Supplementary file 1 [file rbag169_supplementary_data.docx]

**Supplementary Information**

**UV-Grafted Polyitaconic Acid Functionalization of PEEK Promotes Osteoimmunomodulation and Osseointegration with PI3K-AKT-Associated Autophagy Regulation**

Liping Zhou^a,1^, Yang Zhou^b,c,1^, Changyue Liu^b,c,1^, Xiangyu Li^b,c^, Chen Wu^b,c^, Hanling Deng^a^, Wenjiao Zeng^a^, Yongheng Zhang^b,c^, Ying Liu^b,c^, Lvhua Liu^a^, Ang Gao^d***^, Huaiyu Wang^d**^, Yanyan Zheng^a*^

^a^School of Pharmacy and Institute of Pharmacy, North Sichuan Medical College, Nanchong, China

^b^Department of Stomatology, Affiliated Hospital of North Sichuan Medical College, Nanchong, China

^c^Department of Stomatology, North Sichuan Medical College, Nanchong, China

^d^Center for AI-Driven Medical Research, Shenzhen Institutes of Advanced Technology, Chinese Academy of Sciences, Shenzhen, China

^1^ These authors contributed equally to this work

* Corresponding author. E-mail: [yanyzheng@163.com](mailto:yanyzheng@163.com)

** Corresponding author. E-mail: [hy.wang1@siat.ac.cn](mailto:hy.wang1@siat.ac.cn)

*** Corresponding author. E-mail: [ang.gao1@siat.ac.cn](mailto:ang.gao1@siat.ac.cn)

**2. Materials and methods**

**2.1. Materials**

Circular sheets (Ø 15 mm × 2 mm) and rod-shaped samples (Ø 2 mm × 7 mm) were prepared from medical-grade PEEK (Junhua Special Engineering Plastics Products Co., Ltd., China) for *in vitro* and *in vivo* evaluations, respectively. Itaconic acid (ITAc) was obtained from Aladdin Reagent Co., Ltd., China. DMEM, α‑MEM, fetal bovine serum (FBS), 0.25% trypsin, and penicillin‑streptomycin solution were acquired from Hyclone (USA). Phosphate‑buffered saline (PBS), Tris‑HCl buffer, Cell Counting Kit-8 (CCK-8), Western and IP cell lysis buffer, alkaline phosphatase (ALP) assay kit, and BCA protein assay kit were procured from Beyotime Biotechnology (China). TRIzol reagent was obtained from Invitrogen (Thermo Fisher Scientific, USA), and reverse transcription and quantitative real‑time polymerase chain reaction (RT-qPCR) kits were purchased from TaKaRa Bio, Inc. (Japan). Proteinase inhibitor (P1260‑1 mL) and phenylmethylsulfonyl fluoride (PMSF, P0100‑1 mL) were obtained from Beijing Sunlight Biotechnology. The primary antibodies used for western blotting were PI3K (R22768) and p‑PI3K (341468) (1:1000 dilution, Zenbio), AKT (ET1609‑47), p‑AKT (ET1607‑73), p62 (HA721171), and LC3‑I/LC3‑II (ET1701‑65) (1:500 for AKT/p‑AKT/p62, 1:2000 for LC3‑I/LC3‑II, HuaBio). Additionally, 740 Y‑P (GC16157), a potent cell‑permeable PI3K agonist, was acquired from Good Laboratory Practice Bioscience (GLPBIO, USA).

**2.2. PITAc-functionalized PEEK surface**

Before modification, PEEK specimens were sonicated sequentially in ethanol and deionized water and then dried in a vacuum oven. The PEEK sheets were immersed in 1.0 mol/L aqueous ITAc solution and exposed to UV irradiation (1000 W, λ = 365 nm) for 30 min. The reacted specimens were then thoroughly sonicated in deionized water and dried at 37 °C to obtain PITAc-functionalized PEEK, denoted as PEEK-PITAc.

**2.3. Surface characterization**

The surface morphology of samples after being coated with gold was examined by field-emission scanning electron microscopy (FE-SEM, Hitachi S-4200, Japan). Surface roughness and topography were measured by atomic force microscopy (AFM, Bruker Dimension Icon, Germany). Measurements were conducted in tapping mode with ScanAsyst probes under ambient-air conditions, and the scanning region was set as 1 μm × 1 μm. Surface elemental composition and chemical states were analyzed by X-ray photoelectron spectroscopy (XPS, ESCALAB 250Xi, Thermo Fisher Scientific, UK). Surface chemistry was further characterized by diffuse reflectance Fourier transform infrared spectroscopy (DRFTIR, Thermo Scientific Nicolet 5700) with an FT80 accessory. Hydrophilicity was evaluated by static water contact angle measurements using 4 μL deionized water droplets and a contact angle analyzer (DSA100, Kruss, Germany). Surface zeta potential was measured with a SurPASS2 analyzer (Anton Paar GmbH, Austria).

**2.4. Bone-like apatite formation**

PEEK and PEEK-PITAc specimens were immersed in 30 mL simulated body fluid (SBF) and incubated at 37 °C, with SBF refreshed every 3 days. After 30 days of immersion, the specimens were gently rinsed with distilled water, dried under ambient conditions, and analyzed by SEM-EDS to assess surface morphology and elemental composition.

**2.5. Cell culture**

RAW264.7 macrophages, MC3T3-E1 pre-osteoblasts, and human umbilical vein endothelial cells (HUVECs) were obtained from the Cell Bank of the Chinese Academy of Sciences (Shanghai, China). All cell lines were maintained in a humidified incubator at 37 °C under a 5% CO₂ atmosphere. MC3T3-E1 cells were cultured in α-MEM, whereas HUVECs and RAW264.7 macrophages were maintained in high-glucose DMEM. Both media contained with 10% FBS and 1% penicillin-streptomycin, and media were refreshed every 2 days. To induce osteogenic differentiation, MC3T3-E1 culture were supplemented with 100 nM dexamethasone, 10 mM β-glycerophosphate, and 50 μg/mL ascorbic acid (Sigma-Aldrich, USA). Sample/macrophage-conditioned medium (SMCM) was prepared by co-culturing RAW264.7 cells with different specimens for 3 days, collecting the supernatant, centrifuging it at 1500 rpm for 20 min, mixing the supernatant 1:1 with fresh complete medium, and storing it at -80 °C until use.

**2.6. Immune responses of RAW264.7 cells on different samples**

**2.6.1 Cell adhesion**

RAW264.7 macrophages were seeded onto different specimens at a density of 1 × 10⁴ cells per specimen and incubated for 4 and 24 h. The specimens were then rinsed twice with PBS to remove unattached cells and fixed in 2.5% glutaraldehyde solution for 4 h. After fixation, the cells were dehydrated using a graded ethanol series (30%, 50%, 75%, 85%, 95%, and 100%) with each dehydration step lasting 15 min. Finally, the specimens were dried and examined by SEM to evaluate cell adhesion behavior.

**2.6.2 Live/Dead cell assay**

RAW264.7 cells were seeded onto different specimen surfaces at an initial seeding density of 1 × 10⁴ cells per specimen and incubated for 5 days. Cell viability was then assessed with a LIVE/DEAD cell staining (Beyotime, China) and imaged by confocal laser scanning microscopy (CLSM, Olympus, Japan).

**2.6.3 Cell Proliferation**

RAW264.7 macrophages were seeded onto the specimens at an initial seeding density of 1.5 × 10⁴ cells per specimen and cultured for 1, 3, and 5 days, after which cell proliferation was evaluated using a CCK-8. Cells cultured in tissue-culture polystyrene (TCP) wells without material specimens served as the control. At each predetermined time point, CCK-8 reagent was added following the manufacturer’s protocol, and the absorbance at 450 nm was recorded via a microplate reader (Thermo Fisher Scientific, USA).

**2.6.4 Immunoregulation-related gene expression analysis**

RAW264.7 macrophages were cultured on different samples for 3 days. Total RNA was extracted with TRIzol reagent and reverse-transcribed into complementary DNA. RT-qPCR was performed to measure M1-associated genes including inducible nitric oxide synthase (iNOS), cluster of differentiation 86 (CD86), chemokine receptor 7 (CCR7), IL-6, TNF-α, CD11c, IL-1β, M2-associated genes including CD206, CD163, IL-4, IL-10, osteogenesis- and angiogenesis-related genes including bone morphogenetic protein 2 (BMP-2), TGF-β1, platelet-derived growth factor BB (PDGF-BB), vascular endothelial growth factor (VEGF), and autophagy-related genes including autophagy-related gene 5 (ATG5), ATG7, p62, microtubule-associated protein 1 light chain 3α (LC3A), microtubule-associated protein 1 light chain 3β (LC3B), and Beclin 1. Primer sequences are listed in Supplementary Table S1.

**2.6.5 ELISA**

RAW264.7 cells were cultured on different samples for 3 days, after which the supernatant was harvested and centrifuged at 1500 rpm for 20 min. The concentrations of TNF-α, BMP-2, TGF-β1, and VEGF in the supernatant were subsequently quantified using the corresponding ELISA kits (MultiSciences, China) following the manufacturer’s protocols.

**2.7 Molecular mechanisms of immune microenvironment regulation by PEEK-PITAc**

**2.7.1 Western blot analysis of protein expression levels**

RAW264.7 macrophages were directly seeded onto different specimen surfaces, which were assigned to three groups including PEEK, PEEK-PITAc and PEEK-PITAc + 740Y-P. The PEEK-PITAc + 740Y-P group was treated with 740Y-P, a specific PI3K activator. Following 3 days of culture, the cell lysates were prepared on ice utilizing RIPA buffer supplemented with PMSF and Protein Phosphatase Inhibitor (All-in-one, 100x) (Solarbio, Beijing, China), and the supernatant was subsequently harvested by centrifugation. The protein concentration of the supernatant was quantified with a BCA protein assay kit (Solarbio), after which the proteins were denatured by heating at 100 °C. Equal amounts of protein were loaded into each lane of a 10% SDS-polyacrylamide gel alongside a molecular weight marker, followed by electrophoresis. Proteins were then transferred onto polyvinylidene difluoride membranes at 300 mA for 110 min. Membranes were blocked with rapid blocking buffer at room temperature for 20 min and then incubated with primary antibodies overnight at 4 °C. Subsequently, the membranes were incubated with HRP-conjugated secondary antibodies at room temperature in the dark. Protein bands were visualized using an ECL chemiluminescence kit, and grayscale values were quantified using ImageJ software.

**2.7.2 Immunoregulation-related gene expression analysis after treatment with PI3K-AKT agonist 740Y-P**

After 3 days, RT-qPCR was used to quantify M1-associated genes, M2-associated genes, and genes related to osteogenesis, angiogenesis, and autophagy.

**2.8 Effect of immune microenvironment on osteogenic functionality of MC3T3-E1 pre-osteoblasts**

To investigate the regulatory effect of immunomodulation on osteogenic activity, MC3T3-E1 pre-osteoblasts were seeded into 24-well plates and cultured in the SMCM supplemented with osteogenic induction factors.

**2.8.1 Osteogenesis-related gene expression**

After 7 days of culture, the expression levels of osteogenesis-associated genes including ALP, osteocalcin (OCN), osteopontin (OPN), osterix (OSX), bone sialoprotein (BSP), type I collagen (COL-I), receptor activator of nuclear factor kappa-B ligand (RANKL), and SMAD1/4/5/8, and autophagy-associated genes including ATG5, ATG7, p62, LC3A, LC3B, and Beclin 1 were quantified by RT-qPCR. The specific primer sequences for target genes are listed in Supplementary Table S1.

**2.8.2 ALP activity**

After 7 days of culture, MC3T3-E1 pre-osteoblasts were fixed with 4% paraformaldehyde (PFA) for 30 min and subsequently stained with a BCIP/NBT ALP Color Development Kit (Beyotime, China) for 30 min at room temperature in the dark. Optical micrographs were acquired using an inverted microscope. Parallel cultures were lysed in Western and IP lysis buffer and ALP activity and total intracellular protein content in the lysates were quantified using an ALP assay kit and a BCA protein assay kit, respectively, following the manufacturer’s protocols. Finally, ALP activity was normalized to total protein content and expressed as nmol/min/mg protein.

**2.8.3 Extracellular matrix (ECM) mineralization**

ECM mineralization was evaluated via Alizarin Red S staining. After 14 days of culture, MC3T3-E1 pre-osteoblasts were fixed with 4% PFA for 30 min and subsequently stained with 1% Alizarin Red S solution (pH 4.2, Solarbio, China) at room temperature in the dark for 10 min. The cells were then rinsed thoroughly with distilled water to remove unbound dye, and optical micrographs were acquired using an inverted microscope. For quantitative analysis, the adsorbed dye was eluted in 500 μL of 10% cetylpyridinium chloride (Aladdin, China) prepared in 10 mM sodium phosphate buffer (pH 7.0, KESHI, China), and the absorbance of the eluted staining solution at 570 nm was recorded using a microplate reader.

**2.9 Effect of immune microenvironment on angiogenic functionality of HUVECs**

HUVECs were seeded into 24-well plates and cultured in the SMCM to evaluate the regulatory effects of immunomodulation on angiogenesis.

**2.9.1 Angiogenesis-related gene expression**

After 3 days of culture, the expression levels of angiogenesis-associated genes (VEGF, vascular endothelial growth factor receptor 2 (KDR), angiopoietin-1 (ANG-1), BMP-2, fibroblast growth factor (FGF), hypoxia-inducible factor α (HIF-α), CD31, and Smad1/5/8) were analyzed by RT-qPCR. The specific primer sequences for target genes are listed in Supplementary Table S1.

**2.9.2 Cell migration**

A scratch assay was employed to assess the migratory capacity of HUVECs. HUVECs were seeded into 24-well plates at a density of 1 × 10⁵ cells per well and cultured in the SMCM for 3 days until reaching confluence. A scratch was subsequently created using a 200 μL pipette tip, and cell debris was removed by rinsing with PBS. The cells were further incubated in serum-free DMEM for 24 h. Scratch closure was monitored at 0 and 24 h using an inverted optical microscope. The HUVEC migration rate was calculated using the formula (L_0_−L_24_)/L_0_×100%, where L_0_ and L_24_ denote the initial scratch width and the residual scratch width after 24 h of incubation, respectively.

**2.9.3 Angiogenesis**

Matrigel (Corning, USA) was thawed and dissolved overnight at 4 °C prior to use. 10-20 μL Matrigel was added to each well of 24-well plates, evenly spread, and incubated at 37 °C for 60 min to allow gelation. HUVECs were seeded onto the Matrigel surface at a density of 1.5 × 10⁵ cells per well and incubated in the SMCM. After 24 h, tube formation was monitored using an inverted microscope, and total branches length, meshes number, and nodes number were quantified using ImageJ 1.53 software.

**2.10 Evaluation of osteoimmunomodulatory capacities of the samples in direct co-culture**

MC3T3-E1 pre-osteoblasts and RAW264.7 macrophages (both 3 × 10⁴ cells per sample) were directly seeded onto specimen surfaces and cultured in a mixed medium (α-MEM:DMEM = 1:1, 10% FBS, 1% penicillin–streptomycin) for 2 days. The medium was subsequently replaced with mixed medium supplemented with osteogenic induction components and incubated for a further 7 days.

**2.10.1 Osteogenesis- and immunomodulation-related gene expression**

At the predetermined time points, RT-qPCR was used to quantify the expression levels of osteogenesis-associated genes including ALP, Runx2, osterix (OSX), OCN, OPN, BSP, COL-I, and M1 phenotype markers including CCR7, IL-6, TNF-α, IL-1β, M2 phenotype markers including CD163, IL-4, arginase-1 (Arg-1), and osteogenesis- and angiogenesis-associated genes including BMP-2, and VEGF.

**2.10.2 ALP activity**

ALP activity in the direct co-culture system was evaluated qualitatively and quantitatively at the indicated time points.

**2.10.3 Collagen secretion**

At the predetermined time points, the cells were fixed with 4% PFA for 30 min and stained with 0.1% Sirius Red (Sigma, USA) for 18 h. The specimens were subsequently rinsed thoroughly with 0.1 M acetic acid (Aladdin, China) to remove unbound dye, air-dried at room temperature, and imaged. For quantitative analysis, the bound dye was eluted with a 0.2 M NaOH/methanol mixture (1:1 v/v) for 15 min, and the absorbance of the eluted staining solution at 540 nm was measured using a microplate reader.

**2.11. Direct osteogenic functionality of MC3T3-E1 pre-osteoblasts on different samples**

**2.11.1 Cell Adhesion, Live/Dead staining, and proliferation**

MC3T3-E1 pre-osteoblasts were seeded onto different samples and cultured for 4 and 24 h for SEM observation of cell adhesion. Additional cultures were maintained on the specimen surfaces for 5 days, stained with a LIVE/DEAD viability kit, and imaged by CLSM. Cell proliferation was quantified by CCK-8 at days 1, 3, and 5 after seeding.

**2.11.2 Osteogenesis-related gene expression, ALP activity, ECM Mineralization, and collagen secretion**

After 7 and 14 days of culture on different specimens, RT-qPCR was used to quantify osteogenesis-related genes including ALP, OSX, OCN, BSP, and COL-I. ALP activity, ECM mineralization, and collagen secretion were evaluated qualitatively and quantitatively in parallel.

**2.12. Direct angiogenic functionality of HUVECs on different samples**

**2.12.1 Cell Adhesion, Live/Dead staining, and proliferation**

HUVECs were seeded onto different samples and cultured for 4 and 24 h for SEM observation of cell adhesion. Additional cultures were maintained on the specimen surfaces for 3 days, stained with a LIVE/DEAD viability kit, and imaged by CLSM. Cell proliferation was quantified by CCK-8 at days 1, 3, and 5 after seeding.

**2.12.2 Angiogenesis-related gene expression**

After 3 days of culture on different samples, RT-qPCR was used to quantify angiogenesis-related genes including VEGF, KDR, ANG-1, and BMP-2.

**2.13. In vivo evaluation of osseointegration**

**2.13.1 Blood compatibility**

Fresh anticoagulated rat blood (2 mL) was centrifuged at 1500 rpm for 10 min at 4 °C to separate the plasma and white blood cell layer from the red blood cell (RBC) pellet, which was retained. The RBC pellet was gently resuspended in 10 mL of physiological saline, centrifuged at 1500 rpm for 10 min, and the supernatant was discarded. This washing step was repeated three times until the supernatant became colorless and clear. Finally, the RBC pellet was diluted with physiological saline at a 1:49 volume ratio and mixed thoroughly for subsequent use. For control preparations, the positive control was prepared by adding 0.2 mL of 2% erythrocyte suspension to 0.8 mL of distilled water, and the negative control was prepared by mixing 0.2 mL of 2% RBC suspension with 0.8 mL of physiological saline. For the experimental groups, 0.2 mL of 2% RBC suspension was combined with 0.8 mL of physiological saline and the mixture was applied to PEEK and PEEK-PITAc surfaces, respectively. All groups were incubated at 37 °C for 90 min and then centrifuged at 2500 rpm for 5 min. After centrifugation, each group was photographed, and the supernatant was transferred to a 96-well plate. The absorbance at 540 nm was measured using a microplate reader. The hemolysis rate (HR) was calculated using the following formula:

𝐻$R=\frac{OD_{t}-OD_{nc}}{OD_{pc}-OD_{nc}}\times100\%$

where ODt, ODpc, and ODnc represent the absorbance values of the test sample, positive control, and negative control.

**2.13.2 Implantation surgery**

All animal experimental protocols were approved by the Animal Ethics Committee of North Sichuan Medical College (Approval No. 2023376). Eight 12-week-old male Sprague–Dawley (SD) rats (average weight 320 g) were used for the experiments. These were randomly allocated into two groups (n=4 per group), namely the PEEK group and the PEEK-PITAc group. General anesthesia was administered via intraperitoneal injection of 2% pentobarbital sodium at a dosage of 2 mL/kg body weight. Following anesthesia, the surgical site was shaved and rigorously disinfected, and a 10 mm longitudinal incision was made along the lateral side of the extensor mechanism around the knee joint. Blunt dissection of the musculature was performed to expose the femoral condyle. A cylindrical bone defect (7 mm in length, 2 mm in diameter) was created along the longitudinal axis of the femur using a surgical drill. Eight rod-shaped specimens per group were randomly implanted into the femoral condyle defects of 4 individual SD rats. After implantation, the deep musculature and skin were meticulously sutured layer by layer (Supplementary Figure S2). After 8 weeks of implantation, the rats were euthanized, and the femurs with embedded implants were harvested and immediately fixed in 4% paraformaldehyde.

**2.13.3 Micro-CT analysis**

Femoral specimens containing implants were scanned by micro-CT (Pingsheng Healthcare, China). The scanning parameters were set at 80 kV and 50 μA, with a resolution ratio of 35 μm. Following scanning, 3D images were reconstructed using Recon software, and quantitative morphometric analysis was conducted to assess bone regeneration adjacent to the implants. Key bone morphometric parameters were quantified, including bone surface area/bone volume ratio (BS/BV), trabecular thickness (Tb.Th), bone mineral density (BMD), and bone volume fraction (BV/TV).

**2.13.4 Histological Analysis**

Undecalcified femora with implants were embedded in polymethyl methacrylate, sectioned, and then ground to a final thickness of 50 μm. These sections were stained with toluidine blue‑acid fuchsin (Solarbio, China) to characterize the bone‑implant interface and quantify the bone‑implant contact (BIC) ratio. The remaining femoral specimens with implants were decalcified in 10% ethylenediaminetetraacetic acid (Solarbio, China). Following implant explantation, the harvested bone tissues were embedded in paraffin, sectioned, and subjected to standard hematoxylin‑eosin (H&E) and Masson’s trichrome staining (both from Solarbio, China). Histological images were subsequently acquired using an Olympus IX71 microscope (Japan) to evaluate new bone formation adjacent to the implants. Immunohistochemistry (IHC) was performed to detect the expression of OPN, OCN, TNF‑α, and IL‑10. Briefly, paraffin‑embedded sections were deparaffinized with xylene, rehydrated via a graded ethanol series, and treated with 0.3% hydrogen peroxide solution. After blocking with 1% serum (Sigma‑Aldrich, USA), the sections were incubated with primary antibodies targeting OCN, OPN, TNF‑α and IL‑10 (Servicebio, China). After washing, the sections were incubated with corresponding secondary antibodies (Invivogen, USA) and visualized using 3,3’‑diaminobenzidine (DAB) chromogen (Dako, Denmark). Finally, all sections were counterstained with hematoxylin and examined under a light microscope.

**2.14 Statistical Analysis**

All experiments were performed with at least three independent replicates. Statistical analysis was carried out with GraphPad Prism software. Student’s *t*-test was used for comparisons between two groups, and two-way analysis of variance (ANOVA) was used for multiple group comparisons. Statistical significance was defined as *p* < 0.05.

**Figure S1.** Photographs showing the surgical procedures used to implant samples into rat femoral defects.

**Figure S2.** Original gene expression data of RAW264.7 cultured on PEEK and PEEK‑PITAc detected by RT-qPCR. (A) M1-associated genes (CCR7, iNOS, IL-1β, CD11c, TNF-α, CD86, and IL-6). (B) M2-associated genes (CD163, CD206, IL-10, and IL-4), and (C) Osteogenesis- and angiogenesis-related genes (TGF-β1, VEGF, BMP-2, and PDGF-BB) (n = 3 per group). **P* < 0.05 compared to PEEK.

**Figure S3.** Original gene expression data of RAW264.7 cultured on PEEK, PEEK‑PITAc, and PEEK‑PITAc+740Y-P detected by RT-qPCR. (A) M1-associated genes (iNOS, IL-1β, TNF-α, and CD86). (B) M2-associated genes (CD163, CD206, Arg-1, IL-10, and IL-4). (C) Osteogenesis- and angiogenesis-related genes (TGF-β1, VEGF, BMP-2, and PDGF-BB) (n = 3 per group).

**Figure S4.** Original gene expression data of MC3T3-E1 incubated with SMCM from PEEK and PEEK-PITAc detected by RT-qPCR. (A) Osteogenesis-related genes (ALP, COL-I, OCN, OSX, BSP, and RANKL). (B) Smad pathway-related genes (Smad1, Smad4, Smad5, and Smad8) (n = 3 per group). **P* < 0.05 compared to PEEK.

**Figure S5.** Original gene expression data of HUVECs incubated with SMCM from PEEK and PEEK-PITAc detected by RT-qPCR. (A) Angiogenesis-related genes (CD31, VEGFA, KDR, ANG-1, FGF, BMP-2, and HIF-α). (B) Smad pathway-related genes (Smad1, Smad5, and Smad8) (n = 3 per group). **P* < 0.05 compared to PEEK.

**Figure S6.** Original gene expression data of RAW264.7 and MC3T3-E1 co-cultured directly on PEEK and PEEK‑PITAc. (A) M1-associated genes (CCR7, IL-6, IL-1β, and TNF-α). (B) M2-associated genes (CD163, Arg-1, and IL-4) and osteogenesis- and angiogenesis-related genes (BMP-2 and VEGF). (C) Osteogenesis-related gene (ALP, COL-I, OPN, OCN, OSX, RUNX2, and BSP) (n = 3 per group). **P* < 0.05 compared to PEEK.

**(a)**

**(b)**

**Figure S7.** Original gene expression data of MC3T3-E1 cultured directly on PEEK and PEEK‑PITAc for (A) 7 d and (B) 14 d (n = 3 per group). **P* < 0.05 compared to PEEK.

**Figure S8.** **Representative images of the in vitro hemolysis assay.**

**Table S1.** Primers used for RT-qPCR.

| **Genes** | **Primer sequences** |
| --- | --- |
| **Mouse species** | |
| CCR7 | Forward: 5’-TGTACGAGTCGGTGTGCTTC-3’  Reverse: 5’-GGTAGGTATCCGTCATGGTCTTG-3’ |
| CD86 | Forward: 5’-CTGCTCATCATTGTATGTCAC-3’  Reverse: 5’-ACTGCCTTCACTCTGCATTTG-3’ |
| CD11c | Forward: 5’-ACTTCACGGCCTCTCTTCC-3’  Reverse: 5’-CACCAGGGTCTTCAAGTCTG-3’ |
| iNOS | Forward: 5’-GGTGAAGGGACTGAGCTGTTA-3’  Reverse: 5’-TGAAGAGAAACTTCCAGGGGC-3’ |
| IL-1β | Forward 5’-TGGAGAGTGTGGATCCCAAG-3’  Reverse 5’-GGTGCTGATGTACCAGTTGG-3’ |
| IL-4 | Forward: 5’-ATCATCGGCATTTTGAACGAGGTC-3’  Reverse: 5’-ACCTTGGAAGCCCTACAGACGA-3’ |
| CD163 | Forward: 5’-ACATCATGGCACAGGTCAC-3’  Reverse: 5’-TGAGGAAACTGTAAGTCGCTG-3’ |
| CD206 | Forward: 5’-AGACGAAATCCCTGCTACTG-3’  Reverse: 5’-CACCCATTCGAAGGCATTC-3’ |
| Arg-1 | Forward: 5’-CATATCTGCCAAGGACATCG-3’  Reverse: 5’-GGTCTCTTCCATCACTTTGC-3’ |
| TNF-α | Forward: 5’-TAGCCCACGTCGTAGCAAAC-3’  Reverse: 5’-TGTCTTTGAGATCCATGCCGT-3’ |
| IL-6 | Forward 5’-ATAGTCCTTCCTACCCCAATTTCC-3’  Reverse 5’-GATGAATTGGATGGTCTTGGTCC-3’ |
| IL-10 | Forward: 5’-CGGGAAGACAATAACTGCACCC-3’  Reverse: 5’-CGGTTAGCAGTATGTTGTCCAGC-3’ |
| PDGF-BB | Forward: 5’-TCTCTGCTGCTACCTGCGTCT-3’  Reverse: 5’-CAGCCCCATCTTCATCTACGG-3’ |
| TGF-β1 | Forward: 5’-CAGTACAGCAAGGTCCTTGC-3’  Reverse: 5’-ACGTAGTAGACGATGGGCAG-3’ |
| BMP-2 | Forward: 5’-GCTCCACAAACGAGAAAAGC-3’  Reverse: 5’-AGCAAGGGGAAAAGGACACT-3’ |
| VEGF | Forward: 5’- TACCTCCACCATGCCAAGTG-3’  Reverse: 5’- AAGATGTCCACCAGGGTCTC-3’ |
| ATG5 | Forward: 5’-GATGCGGTTGAGGCTCAC-3’  Reverse: 5’-CTGTCATTCTGCAGTCCCATC-3’ |
| ATG7 | Forward: 5’-AGCCTGTTCACCCAAAGTTC-3’  Reverse: 5’-CATGTCCCAGATCTCAGCAG-3’ |
| LC3B | Forward: 5’-GATAATCAGACGGCGCTTGC-3’  Reverse: 5’-TCTCACTCTCGTACACTTCGG-3’ |
| LC3A | Forward:5’-ACAGCATGGTGAGCGTCTC-3’  Reverse:5’-AGGTTTCTTGGGAGGCGTAG-3’ |
| P62 | Forward: 5’-AGCTGCTCTTCGGAAGTCAG-3’  Reverse: 5’-CTCCATCTGTTCCTCTGGCTG-3’ |
| Beclin1 | Forward: 5’-CAGTACCAGCGGGAGTATAGTGA-3’  Reverse: 5’-TGTGGAAGGTGGCATTGAAGA-3’ |
| ALP | Forward: 5’-CCAGCAGGTTTCTCTCTTGG-3’  Reverse: 5’-GGGATGGAGGAGAGAAGGTC-3’ |
| OSX | Forward: 5’-GCTGCCTACTTACCCGTCTG-3’  Reverse: 5’-AGGTTTGCCTGCACCACTC-3’ |
| RUNX2 | Forward: 5’-CCCAGCCACCTTTACCTACA-3’  Reverse: 5’-TATGGAGTGCTGCTGGTCTG-3’ |
| OPN | Forward: 5’-TCTGATGAGACCGTCACTGC-3’  Reverse: 5’-AGGTCCTCATCTGTGGCATC-3’ |
| OCN | Forward: 5’-GGACCATCTTTCTGCTCACTCTG-3’  Reverse: 5’-TTCACTACCTTATTGCCCTCCTG-3’ |
| BSP | Forward: 5’-CAGGGAGGCAGTGACTCTTC-3’  Reverse: 5’-AGTGTGGAAAGTGTGGCGTT-3’ |
| COL-I | Forward: 5’-GAGCGGAGAGTACTGGATCG-3’  Reverse: 5’-GTTCGGGCTGATGTACCAGT-3’ |
| RANKL | Forward: 5’-GAAACATCGGGAAGCGTACC-3’  Reverse: 5’-GTACGTCGCATCTTGATCCG-3’ |
| Smad1 | Forward: 5’-GTATGAGCTTTGTGAAGGGC-3’  Reverse: 5’-TAAGAACTTTATCCAGCCACTGG-3’ |
| Smad4 | Forward: 5’-CTCCAGCTATCAGTCTGTCAG-3’  Reverse: 5’- CCCGGTGTAAGTGAATTTCAAT-3’ |
| Smad5 | Forward: 5’-TCATCATGGCTTTCATCCCACC-3’  Reverse: 5’-GCTCCCCAACCCTTGACAAA-3’ |
| Smad8 | Forward: 5’-ACTGGTGTTCTGTTGCCTACTAC-3’  Reverse: 5’-AAGATGCTGCTGTCGCTCAC-3’ |
| GAPDH | Forward: 5’-ACCCAGAAGACTGTGGATGG-3’  Reverse: 5’-CACATTGGGGGTAGGAACAC-3’ |
| **Human species** | |
| VEGFA | Forward: 5’-CTTGCCTTGCTGCTCTACCT-3’  Reverse: 5’-GCAGTAGCTGCGCTGATAGA-3’ |
| KDR | Forward: 5’-GTGATCGGAAATGACACTGGAG-3’  Reverse: 5’-CATGTTGGTCACTAACAGAAGCA-3’ |
| CD31 | Forward:5’-CAACGAGAAAATGTCAGA-3’  Reverse:5’-GGAGCCTTCCGTTCTAGA-3’ |
| ANG-1 | Forward:5’-GTGCTGGGTCTGGGTCTGAC-3’  Reverse:5’-GGCCTTGATGCTGCGCTTG-3’ |
| FGF | Forward:5’-CTGTACTGCAAAAACGGG-3’  Reverse:5’-AAAGTATAGCTTTCTGCC-3’ |
| BMP-2 | Forward:5’-TGGCCCACTTGGAGGAGAAACA-3’  Reverse:5’-CGCTGTTTGTGTTTGGCTTGACG-3’ |
| HIF-α | Forward:5’-CCATGTGACCATGAGGAAAT-3’  Reverse:5’-CGGCTAGTTAGGGTACACTT-3’ |
| Smad1 | Forward: 5’-TGCCACTCAACGCCACTTTT-3’  Reverse: 5’-TCATAAGCAACCGCCTGAACAT-3’ |
| Smad5 | Forward: 5’-ACGTCAATGGCCAGCTTGTT-3’  Reverse: 5’-TCCAACGGCTTTAGCTCATGA-3’ |
| Smad8 | Forward: 5’-ATCTTTGTGCAGAGCCGGAA-3’  Reverse: 5’-TCCTGGCGATGATACTCAGCA-3’ |
| β-actin | Forward: 5’-GCACTCTTCCAGCCTTCCTT-3’  Reverse: 5’-AATGCCAGGGTACATGGTGG-3’ |

**Table S2.** Relative surface concentrations of C1s and O1s and and the O1s/C1s ratio for PEEK and PEEK-PITAc.

| Sample | Relative concentration (%) | | O1s/C1s |
| --- | --- | --- | --- |
|  | C1s | O1s |  |
| PEEK | 86.19 | 13.81 | 0.160 |
| PEEK-PITAc | 74.70 | 25.30 | 0.339 |

**Table S3.** Relative contents of the different C species and O species on PEEK and PEEK-PITAc.

| Spectra | B.E.（eV） | Attribution | Relative content （%） | |
| --- | --- | --- | --- | --- |
|  |  |  | PEEK | PEEK-PITAc |
| C1s | 284.8 | C-C/C-H | 68.93 | 62.80 |
|  | 286.2 | C-O | 16.98 | 18.42 |
|  | 287.2 | C=O | 9.25 | 5.51 |
|  | 289.1 | COOH | - | 10.98 |
|  | 291.5 | π→π* | 4.84 | 2.29 |
| O1s | 531.4 | O=C | 29.76 | 10.44 |
|  | 532.1 | O=C-OH | - | 34.82 |
|  | 533.3 | O-C/O=C-OH | 70.24 | 54.74 |

**Table S4.** EDS elemental analysis of PEEK-PITAc after immersion in SBF for 30 days.

| Element | Wt% | At% |
| --- | --- | --- |
| C | 8.44 | 16.55 |
| O | 29.6 | 43.59 |
| P | 19.91 | 15.14 |
| Ca | 42.05 | 24.72 |
| Total | 100 |  |
